# Positive Myelin Oligodendrocyte Glycoprotein Antibodies in Isolated Optic Neuritis in a 14-Year-Old Child

**DOI:** 10.7759/cureus.61371

**Published:** 2024-05-30

**Authors:** Madhawi J Albuainain, Ali Alfehaid, Raafat Hammad Seroor Jadah

**Affiliations:** 1 Internal Medicine, Bahrain Defence Force Hospital, Riffa, BHR; 2 Internal Medicine, King Hamad University Hospital, Muharraq, BHR; 3 Paediatrics and Child Health, Bahrain Defence Force Hospital, Riffa, BHR

**Keywords:** myelin oligodendrocyte glycoprotein (mog), blind, steroid treatment, steroid, demylination, mog antibody-associated disease, acute optic neuritis, optic neuritis

## Abstract

Optic neuritis (ON) is a rare condition in the pediatric age group. Patients with optic neuritis can manifest with a wide range of drops in vision, ranging from mild loss to complete loss of vision. Knowing the cause of optic neuritis is an important point that will affect management and prognosis. Anti-myelin oligodendrocyte glycoprotein (anti-MOG) antibody is an autoantibody that causes demyelination of the central nervous system (CNS). Treatment with a high dose of IV steroids followed by oral steroids is the best regimen that shows a favorable vision outcome. We aim to report this case of isolated optic neuritis with a positive anti-myelin oligodendrocyte glycoprotein antibody to highlight the prognosis of myelin oligodendrocyte glycoprotein disease with isolated optic neuritis and how early diagnosis and treatment can affect the visual outcome.

## Introduction

Anti-myelin oligodendrocyte glycoprotein (anti-MOG) antibody is an autoantibody that induces inflammatory demyelinating lesions in the central nervous system (CNS) in diseases such as optic neuritis (ON), encephalitis, and myelitis [1]. In addition, anti-MOG antibodies can be found in demyelinating diseases of the central nervous system, such as multiple sclerosis (MS), myelin oligodendrocyte glycoprotein antibody disease (MOGAD), and neuromyelitis optica spectrum disorder (NMOSD) [2]. Myelin oligodendrocyte glycoprotein antibody disease (MOGAD) is a recently emerging concept characterized by the presence of anti-myelin oligodendrocyte glycoprotein (MOG) antibodies in the serum or cerebrospinal fluid (CSF) [3]. The most common presenting symptom of MOGAD is optic neuritis, followed by acute disseminated encephalomyelitis (ADEM) [4]. Optic neuritis associated with multiple sclerosis usually presents with painful vision loss that is progressive over a period of a few days. Having painless vision loss or progressive vision loss for over a week should be investigated for other rare demyelinating diseases such as myelin oligodendrocyte glycoprotein antibody disease (MOGAD) and neuromyelitis optica spectrum disorder (NMOSD) [5]. Once the diagnosis of optic neuritis is established, searching for the exact cause is highly important as it will determine the management and prognosis of the condition [6]. We aim to report this case of isolated optic neuritis with a positive anti-MOG antibody, to highlight the prognosis of seropositive optic neuritis and how early diagnosis and treatment can affect the visual outcome.

## Case presentation

A 14-year-old male, with no medical conditions, visited the ophthalmology clinic due to a sudden decrease in vision in his right eye over the past week. The loss of vision was sudden and not preceded by trauma or infection. The patient reported that the vision loss started gradually for over a week. The vision loss started with blurring of vision on the first day and then turned to black vision at the end of the week. It was not associated with eye pain. There is no history of headache, abnormal movement, or fever.

On physical examination, the child has normal neurological examination, tone, power, and reflexes. All cranial nerve (CN) examinations were normal, except for the right eye, which shows a sluggish reaction to light and complete loss of vision. The left eye was reactive to light with no affection for vision. Cerebellar examinations were normal. The rest of the systemic examination is also unremarkable. Eye examination shows right optic disc edema, which suggests optic neuritis.

**Figure 1:**
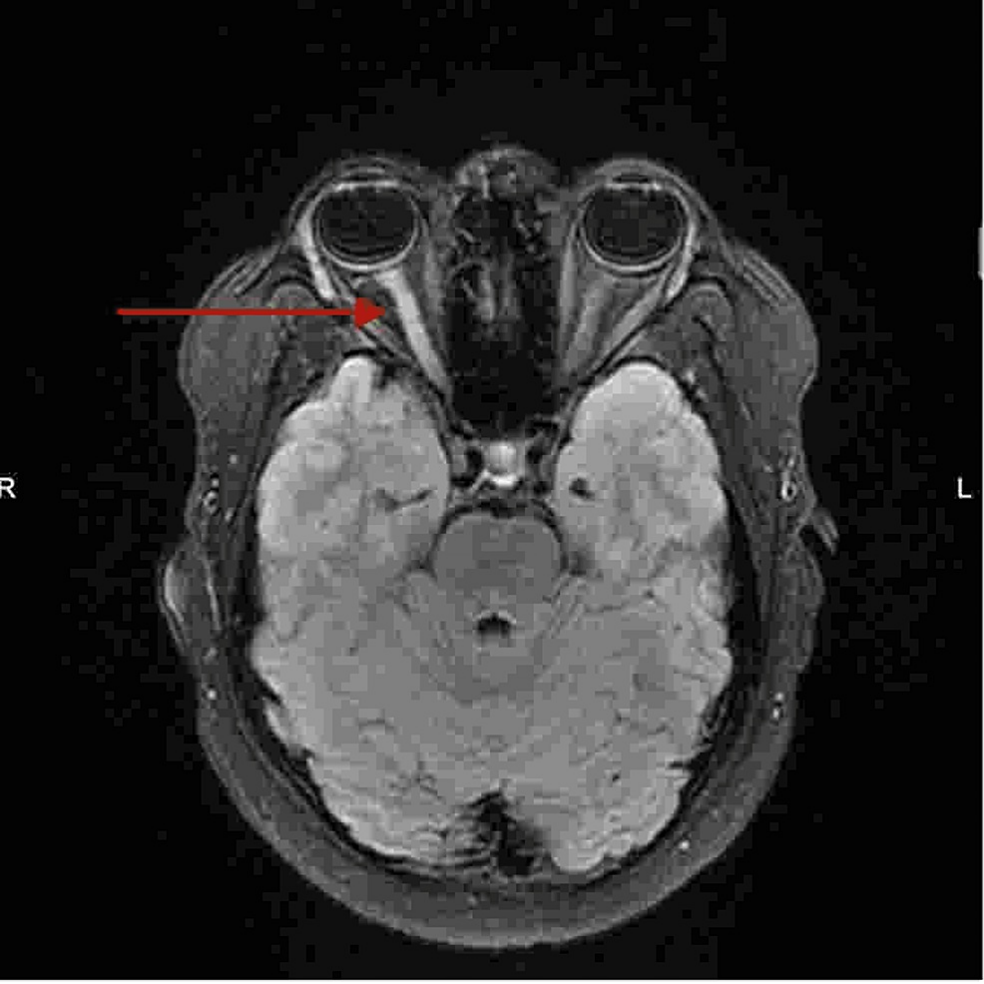
MRI of the brain and orbit T2/FLAIR signal of the aforementioned segments of the right optic nerve is also increased compared to the left side with increased contrast enhancement more prominent at the pre-chiasmatic segment (arrow). MRI: magnetic resonance imaging, FLAIR: fluid-attenuated inversion recovery

The patient underwent magnetic resonance imaging (MRI) of the brain and orbit (Figure 1). The right optic nerve shows increased contrast enhancement compared to the normal left side. The constellation of findings is suggestive of right-sided optic neuritis. Also, visual evoked potential study was done for the patient, which showed evidence of bilateral optic neuropathy more on the right side (prolong bilateral latencies with medium amplitude). Based on the patient's clinical condition, anti-myelin oligodendrocyte glycoprotein (anti-MOG) antibody was requested and confirmed the high value of the ratio (1:80). Cerebrospinal fluid is negative for oligoclonal band (Table 1).

**Table 1:** CSF analysis results CSF: cerebrospinal fluid, IgG: immunoglobulin G, MOG: myelin oligodendrocyte glycoprotein, IFT: immunofluorescence test

CSF analysis	Result	Unit	Reference range
Oligoclonal bands (CSF)	Negative	-	Negative
Albumin (CSF)	133	mg/L	100-300
IgG (CSF)	22.30	mg/L	<34
Oligoclonal bands (serum)	Negative	-	-
Albumin (serum)	47.5	g/L	35-52
IgG (serum)	14.3	g/L	-
Albumin ratio (CSF/serum)	2.8	-	≤4.9
IgG intrathecal synthesis	Not detectable	%	Not detectable
Autoimmune serology MOG antibodies (IgG) (IFT) (serum)	1:80	Titer	<1:10

Comprehensive laboratory investigation and imaging suggest the presence of optic neuritis. As a result, the patient started on a pulse dose of IV methylprednisolone sodium succinate (30 mg/day) for five days. The patient regained vision in 48 hours. This pulse IV steroid was followed by a tapering dose of oral steroid for a two-week duration. He was discharged with follow-up after one week in the clinic with an oral steroid 15 mg/5 mL syrup bottle. The patient presented to the follow-up clinic with a complete recovery of vision without any impairment.

## Discussion

Optic neuritis is a rare condition in childhood that accounts for 25% of pediatric acute demyelinating syndrome [1]. Myelin oligodendrocyte glycoprotein disease most commonly affects the optic nerve, causing optic neuritis [4]. MOG antibody-positive patients will present in 54%-61% of patients initially with optic neuritis [7]. Optic neuritis (ON) refers to inflammatory demyelinating lesions of the optic nerve [1] that usually result from an autoimmune response. The pathophysiology of optic neuritis starts with inflammation that, if left untreated, can lead to secondary death of the nerve [8]. Two types of optic neuritis have been identified: typical ON, which is associated with multiple sclerosis (MS), and atypical ON, which is associated with MOG antibody-associated disease and neuromyelitis optica spectrum disorder (NMOSD). Differentiating between the two types is important since they differ in prognosis and treatment choice. Atypical optic neuritis is usually associated with more severe vision loss than the typical type [6,9].

Our patient presented with sudden unilateral complete loss of vision with rapid onset, which was not associated with other neurological symptoms. Magnetic resonance imaging (MRI) showed increased T2 hyperintensity of the right optic nerve, a typical finding in optic neuritis [10,11]. The chiasm and retro-chiasmatic pathways are spared [12]. In the case of MOGAD patients, MRI findings of the optic nerve are more edematous than the typical ON with extensive inflammatory lesions [13].

Treating optic neuritis aims to decrease the inflammation that affects the optic nerve [14]. Patients with anti-MOG antibody-positive optic neuritis respond well to steroid therapy. Responding to treatment is affected by two main points: the type of steroid and the dose [15,16]. Treatment with IV steroids at a high dose (30 mg/kg/day) is linked to fast recovery and better outcomes [17,18]. However, MOGAD patients who are refractory to initial treatment with glucocorticoids or show weak response to the treatment can have plasma exchange, which can be administered every other day for a total of 5-7 exchanges in total. A randomized trial done in 1999 for patients with central nervous system (CNS) inflammatory demyelinating disease who were refractory to IV glucocorticoids showed that plasma exchange can result in moderate to greater improvement in neurological disability for such patients [19]. Moreover, intravenous immune globulin (IVIG) can be an alternative option in children with MOGAD and administered as a total dose of 2 g/kg divider over 2-5 days [20].

## Conclusions

Having a child with complete vision loss is devastating to the child and the family. It is important to suspect common causes as well as rare causes of complete vision loss. Isolated optic neuritis with a positive anti-MOG antibody is rarely seen in the pediatric age group. Hence, early treatment with a pulse steroid has an important effect on the course and outcome of such cases. Therefore, it is important to have a high index of suspicion in patients who have been presenting with vision loss for more than a week.
